# Calcium Electroporation versus Electrochemotherapy with Bleomycin in an In Vivo CAM-Based Uveal Melanoma Xenograft Model

**DOI:** 10.3390/ijms25020938

**Published:** 2024-01-11

**Authors:** Theodora Tsimpaki, Ralitsa Anastasova, Hongtao Liu, Berthold Seitz, Nikolaos E. Bechrakis, Utta Berchner-Pfannschmidt, Miriam M. Kraemer, Miltiadis Fiorentzis

**Affiliations:** 1 Department of Ophthalmology, University Hospital Essen, University of Duisburg-Essen, Hufeland Str. 55, 45147 Essen, Germany; theodora.tsimpaki@uk-essen.de (T.T.); ralitsa.anastasova@uk-essen.de (R.A.); hongtao.liu@uk-essen.de (H.L.); nikolaos.bechrakis@uk-essen.de (N.E.B.); utta.berchner-pfannschmidt@uk-essen.de (U.B.-P.); miriam.kraemer@uk-essen.de (M.M.K.); 2 Department of Ophthalmology, Saarland University Medical Center, Kirrberger Str. 100, 66421 Homburg, Germany; berthold.seitz@uks.eu

**Keywords:** uveal melanoma, electroporation, electrochemotherapy, calcium electroporation, chick chorioallantois membrane assay, uveal melanoma xenografts, bleomycin

## Abstract

Despite recent advancements in the diagnosis and treatment of uveal melanoma (UM), its metastatic rate remains high and is accompanied by a highly dismal prognosis, constituting an unmet need for the development of novel adjuvant therapeutic strategies. We established an in vivo chick chorioallantoic membrane (CAM)-based UM xenograft model from UPMD2 and UPMM3 cell lines to examine its feasibility for the improvement of selection of drug candidates. The efficacy of calcium electroporation (CaEP) with 5 or 10 mM calcium chloride (Ca) and electrochemotherapy (ECT) with 1 or 2.5 µg/mL bleomycin in comparison to monotherapy with the tested drug or electroporation (EP) alone was investigated on the generated UM tumors. CaEP and ECT showed a similar reduction of proliferation and melanocytic expansion with a dose-dependent effect for bleomycin, whereas CaEP induced a significant increase of the apoptosis and a reduction of vascularization with varying sensitivity for the two xenograft types. Our in vivo results suggest that CaEP and ECT may facilitate the adequate local tumor control and contribute to the preservation of the bulbus, potentially opening new horizons in the adjuvant treatment of advanced UM.

## 1. Introduction

Melanoma is a relatively rare tumor arising from melanocytes located at various anatomic locations, including skin, mucous membrane, ocular tract, and rarely from unknown primary sites [1]. The uveal tract, which contains melanocytes, is the heavily vascularized layer of the globe encompassing the iris (4%), ciliary body (6%), and choroid (90%) [2]. UM is a relatively rare disease primarily found in the Caucasian population and represents the most common primary intraocular malignant tumor in adults, with an incidence of approximately 2–8 cases per million per year [3,4]. The host susceptibility factors for UM include fair skin, light eye color, inability to tan, ocular or oculodermal melanocytosis, cutaneous, iris or choroidal nevus, and *BRCA1*-associated protein 1 mutation [5,6]. UM is more commonly seen in older age groups, and the median age of diagnosis is 59–62 years in the United States and Europe, where there is a prominent Caucasian population [2,7,8].

UM has a high tendency to metastasize hematogenously, commonly including the liver (89%), lungs (29%), and bone (17%), resulting in high mortality [1,5,6,7,8,9,10]. The metastasis-associated mortality rate in patients with UM is approximately 50% within 10 years of diagnosis, irrespective of the type of treatment [3,9,10,11]. Various clinical, histopathological, and cytogenetic features of UM can identify patients with a higher risk of developing metastasis, who may avail from appropriate adjuvant treatments. Characteristics, such as large tumor size, extraocular extension, high mitotic activity, and an epithelioid cell type, constitute significant prognostic factors, whereas the spindle cell type is associated with low metastatic risk [6,7]. Aberrations in chromosome 1, 3, 6, and 8 determine the survival in patients with UM [11,12,13,14,15,16]. Chromosome 3 loss, 8q gain, 1p loss, and 6q loss are associated with poor prognosis. Partial aberrations on chromosome 3 and isodisomy have also been reported, both of which have a metastatic potential [17,18]. Furthermore, frequently mutated genes in UM are *GNAQ* and *GNA11*, which are involved in the Gα signaling pathway and are mutually exclusive in most tumors. Other frequently mutated genes entail *BAP1, SF3B1, and EIF1AX* [19,20,21,22].

A standardized therapeutic regimen has not yet been established for the consolidation of a common protocol for the conservative and surgical treatment of UM patients, depending on various tumor characteristics or the progression of the disease. The current options for UM include brachytherapy or teletherapy and tumor excision via transscleral resection, endoresection, or enucleation [2,7]. In spite of the initial satisfactory local control, the metastatic and the mortality rate remain high. The collaborative uveal melanoma study group reported that the death rate following the report of melanoma metastasis was 80% at 1 year and 92% at 2 years. Overall survival after metastasis did not vary by baseline size of primary tumor nor treatment for metastasis. Long-term survival after diagnosis of metastasis was uncommon [9]. Therefore, the investigation of new reliable in vivo models and their use in the development of novel therapeutic strategies is urgently required.

The chick embryo chorioallantoic membrane (CAM) constitutes a suitable time- and cost-efficient preclinical model to study different cancer-associated processes, including angiogenesis, cell invasion, metastasis profiling, and tumor progression, or to screen potential therapeutic modalities and agents [23]. During avian development, the mesodermal layers of the allantois and chorion fuse to form the CAM. This structure rapidly expands, generating a rich vascular network that provides an interface for gas and waste exchange [24]. The embryo is not fully immunocompetent until day 18, although a non-specific inflammatory reaction can occur after day 15 [23,24,25,26]. Furthermore, tumors develop much faster in the CAM, in approximately 3–4 days, compared to 3–4 weeks in the mouse models, due to the fact that the oxygen and nutrients are provided by developing blood vessels and due to its natural immunodeficiency [24]. Thus, the chick embryo represents a reliable model for transplantations from different cells, tissues, and species, without immune responses, allowing the examination of the generated tumor grafts, their growth and metastatic ability, angiogenetic molecules, drugs delivery, and their respective efficacy.

Thermal tumor ablation techniques, including radiofrequency, laser, high-intensity focused ultrasound, and cryoablation are routinely used in the therapy of various cancer entities. However, they comprise thermal techniques, which may be affected by the heat sink effect, leading to incomplete ablation and thermal injuries of non-targeted surrounding structures. High-voltage pulsed electric field can induce the formation of aqueous nanopores in the cell membrane and an increased plasma membrane permeability [27,28]. This phenomenon is also known as electroporation. Reversible electroporation and therefore transient cell permeability enables the diffusion of low or non-permeable drugs, such as chemotherapeutic agents bleomycin and cisplatin, into the cell cytosol [29]. This conjunction, also described as electrochemotherapy, allows the application of significant lower chemotherapeutic doses due to the locally potentiated cytotoxic effect of the agents [30]. Furthermore, EP in combination with calcium ions was proposed as a novel anticancer treatment with emphasis on the avoidance of the harmful systemic effects of chemotherapeutic drugs [31,32,33]. The increased influx of calcium ions induces an intracellular calcium overload, which can cause cell death of cancer cells without significantly damaging surrounding structures and cells. Furthermore, disruptions in calcium homeostasis have been observed in cancer cells and can lead to detrimental effects, including mitochondrial dysfunction and acute ATP depletion [31,32,34]. Although ECT has already been established as an adjuvant therapeutic option for solid tumors, including cutaneous melanoma, the modality has not been adequately researched for its application and potency in UM.

In this study, we established and evaluated the suitability of an in vivo model based on the CAM assay after transplantation of tumors generated from the UPMD2 and UPMM3 cell lines [35,36]. In constant pursuance of new antitumor applications, the tumor grafts on the CAM were treated with EP in combination with 1 or 2.5 µg/mL bleomycin or with 5 or 10 mM calcium to investigate the feasibility and efficacy of these modalities for UM. Thus, this methodological novelty improves reproducibility in the field of in vivo experimentation, emphasizing the CAM assay as an alternative to rodent xenograft models, and underlines the in vivo potency of ECT and CaEP in the treatment of UM tumors.

## 2. Results

### 2.1. Size Characterization of the Tumor Xenografts

The mortality of the eggs did not vary significantly between the two cell lines and was approximately 49% from the first development day until the dissection. A total of 235 eggs were implanted with uveal melanoma UPMD2 and UPMM3 xenografts, whereas 53 UPMD2 and 56 UPMM3 tumor nodules could be dissected on ED18. The experiments were performed in four to nine independent biological replicates on different dates. The UPMM3 cell line formed less pigmented white to brown nodules on the surface of the CAM, while the UPMD2 grafts showed strongly pigmented dark brown tumors, facilitating the visualization and assessment during the development and treatment. The formation of solid nodules with a surrounding vascular star was considered a sign of successful implantation and growth. The generated tumors were precisely measured regarding the length and width after their excision.

**Figure 1:**
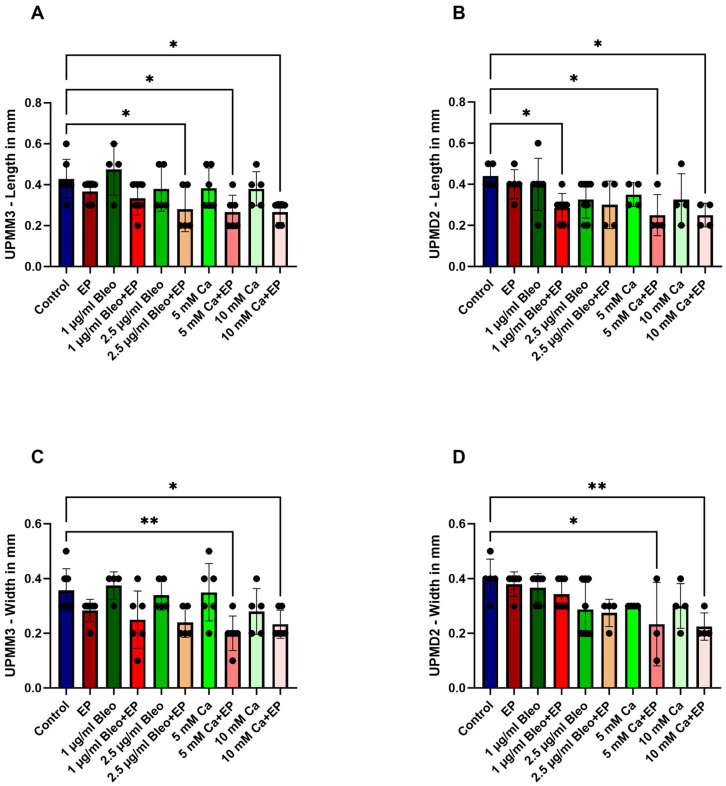
Size characterization of the implanted UPMM3 and UPMD2 tumors in the CAM assay after treatment with electroporation and various dosages of bleomycin and calcium chloride in comparison to the untreated control group. (**A**,**B**): Differences in length for the implanted grafts after different EP conditions. A significant decrease of length was observed after EP in combination with 2.5 µg/mL bleomycin in the UPMM3 tumors, with 1 µg/mL bleomycin in the UPMD2 tumors, as well as with 5 and 10 mM calcium chloride in both cell lines. (**C**,**D**): Width of the generated tumors according to each tested EP condition. An apparent reduction of width was documented in the grafts after application of 5 or 10 mM calcium chloride prior to EP for both UPMM3 and UPMD2 tumors. Statistical analysis was performed using a one-way ANOVA and Dunnet’s multiple comparison test. Significance levels are indicated with * *p* < 0.05, ** *p* < 0.01.

In the control group, the implanted grafts showed a mean length of 0.42 (±0.09 SD) cm and 0.44 (±0.05 SD) cm and a mean width of 0.35 (±0.07 SD) cm and 0.4 (±0.07 SD) cm for the UPMM3 and UPMD2 cell lines, respectively (Figure 1A–D). The ECT combined with 2.5 µg/mL bleomycin had a statistically significant effect on the length of the UPMM3 grafts compared to the control (*p* = 0.0477), whereas a similar effect was achieved after application of solely 1 µg/mL of the same chemotherapeutic agent for the UPMD2 implants (*p* = 0.038) (Figure 1A,B). Furthermore, an apparent reduction of length and width could be achieved after the intratumoral injection of calcium chloride prior to EP. A statistically significant reduction was documented with length parameters of 0.26 (±0.08 SD) cm and 0.026 (±0.05 SD) cm in UPMM3 nodules and width of 0.25 (±0.1 SD) cm and 0.25 (±0.05 SD) cm in UPMD2 tumors after administration of 5 and 10 mM Ca in combination with EP, respectively (Figure 1A–D).

### 2.2. Histological Assessment of the Tumor Xenografts

The specimens were further assessed and measured after histological staining regarding their respective dimensions in each treatment group. A significant shrinkage of approximately 25% was documented in comparison to the excised samples after paraffinization for all tested conditions.

**Figure 2:**
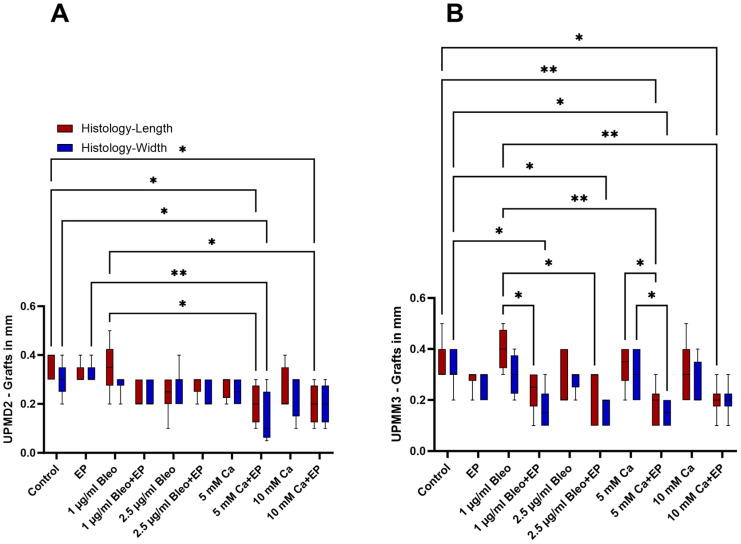
Comparison of the length and width of the UPMD2 and UPMM3 tumors based on the measurements after histological assessment. (**A**,**B**): Presentation of the differences in the grafts’ length (red) and width (blue) in each treatment setting. (**A**): A statistically significant reduction of the size parameters was detected in the groups treated with EP and 5 or 10 mM Ca in the UPMD2 tumors. (**B**): Significant decrease of the size parameters was observed in the ECT group with 1 or 2.5 µg/mL bleomycin as well as after CaEP with both tested concentrations of calcium, 5 and 10 mM. Statistical analysis was performed using a two-way ANOVA and Tukey’s multiple comparisons test. Significance levels are indicated with * *p* < 0.05, ** *p* < 0.01.

In the UPMD2 tumors, the size of the control group as well as of the group treated with EP alone and 1 µg bleomycin alone demonstrated higher values, leading to a statistically significant difference, especially in comparison to the tumors, which underwent EP with either 5 or 10 mM Ca (*p* = 0.0445) (Figure 2A). Specifically, the histologically measured length and width of the UPMD2 xenografts in the control group were calculated as 0.36 (±0.055 SD) cm and 0.3 (±0.071 SD) cm accordingly, whereas these parameters were 0.2 (±0.082 SD) cm and 0.138 (±0.111 SD) cm after application of EP with 5 mM Ca. An apparent diminution of the UPMD2 nodules after EP and bleomycin alone could not be observed in comparison to the untreated control.

A greater variation of the size parameters was observed among the UPMM3 tumors after histological evaluation. Both tested dosages of bleomycin showed a higher efficacy with a statistically significant reduction of width, particularly after ECT with 2.5 µg/mL bleomycin (Figure 2B). In opposition to UPMD2, calcium alone showed a less apparent effect on the achieved shrinkage of the UPMM3 tumors. When EP was combined with both concentrations of calcium, a significant decrease in the length (*p* = 0.005) and width (*p* = 0.01) was noted for the 5 mM and in length for the 10 mM Ca concentration (*p* = 0.01) in comparison to the control group. The mean length and width decreased significantly from 0.357 cm (±0.079 SD) and 0.314 cm (±0.069 SD) in the control group to 0.183 cm (±0.075 SD) and 0.15 cm (±0.055 SD) after EP with 5 mM Ca and to 0.2 cm (±0.063 SD) after EP with 10 mM Ca.

**Figure 3:**
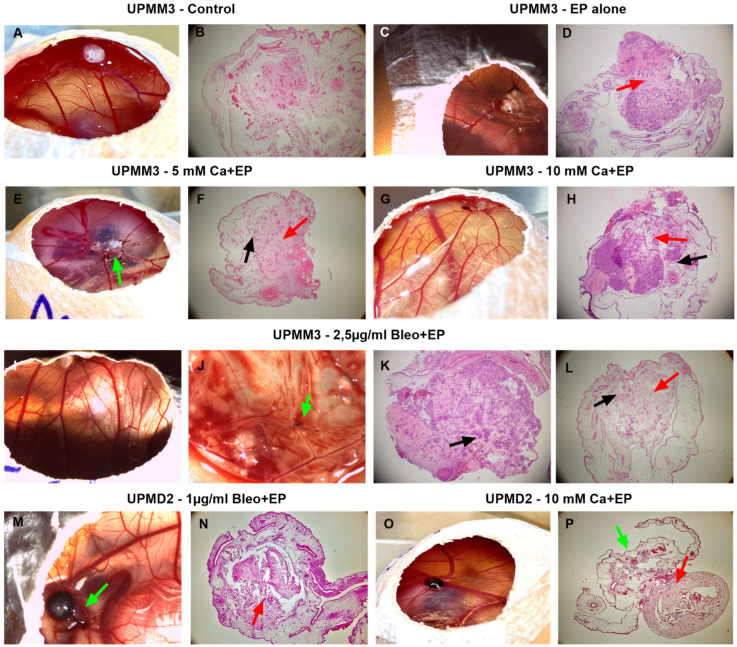
Exemplary images of the UPMM3 and UPMM3 tumors on the CAM before the dissection as well as the hematoxylin and eosin staining after various treatment conditions with electroporation. (**A**,**B**): Lightly pigmented nodular untreated UPMM3 tumor on the CAM in the proximity of a vascular bifurcation on ED 18 before dissection (**A**). H&E staining of the tumor with prominent intratumoral vasculature (**B**). (**C**,**D**): Light brown whitish UPMM3 tumor xenograft on the CAM surface on ED 18 with the formation of a vascular star on the engraftment site after treatment with EP alone on ED14, (**C**) and its respective H&E staining, where there are signs of eminent dissociation centrally (red arrow) (**D**). (**E**,**F**): Examples of UPMM3 tumors on ED 18 before dissection priorly treated with 5 mM Ca and EP and the respective H&E staining of image (**E**). Signs of dissociation of the tumor (red arrow), formation of cell aggregates intratumorally (black arrow), and migration of pigmented cells in the periphery (green arrow). (**G**,**H**): Unpigmented UPMM3 tumor after intratumoral injection of 10 mM Ca and subsequent EP and H&E staining (**I**–**L**): Images of unpigmented UPMM3 tumor nodule after treatment with 2.5 µg/mL bleomycin and EP and H&E staining images of treated grafts. Signs of intravascular invasion and migration of pigmented tumor cells (green arrow) are seen in the lower CAM (**J**). Formation of globular cell aggregates and irregular central tumor cell distribution intratumorally (black arrow) (**K**,**L**). (**M**,**N**): Strongly pigmented UPMD2 tumor xenograft on ED 18 after application of 1 µg/mL bleomycin combined with EP and its H&E staining. Peripheral loss of adherence with intravascular migration of pigmented UM cells (green arrow) (**M**) and central dissociation of the tumor graft (**N**). (**O,P**): Intensely pigmented UPMD2 tumor on ED 18 with its H&E staining after administration of 10 mM calcium chloride and application of EP. Formation of a vascular star at the basis of the graft (**O**). Dissociation of the tumor core with intense pigmentation in the marginal area (red arrow) as well as migration of pigmented tumor cells in the proximal CAM area underneath the graft (green arrow) (**P**). 4× magnification of the H&E staining.

**Figure 4:**
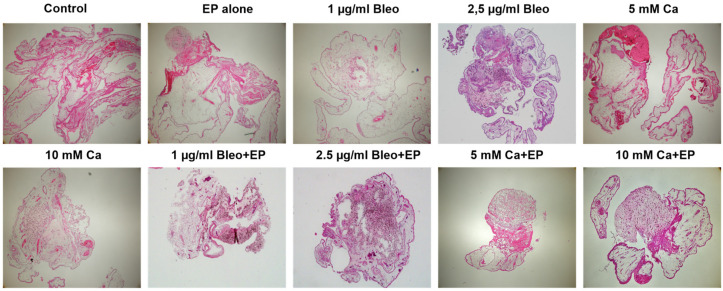
Hematoxylin and eosin staining of UPMD2 tumors after excision from the CAM in the control group as well as in the groups treated with EP alone, 1 and 2.5 µg/mL bleomycin alone or in combination with EP, or 5 and 10 mM Ca alone or in combination with EP. Regressive cellular changes and an intense increase in pigmentation were more apparent in the groups treated with EP and either bleomycin or calcium, with a prominent dose-dependent effect for the ECT group with bleomycin. 4× magnification of the H&E staining.

The hematoxylin and eosin staining and the brightfield microscopy of both cell line tumors revealed the integration of the tumor xenografts into the CAM, signs of vertical growth, as well as the intratumoral vascular growth, especially in the untreated group (Figure 3A,B). In addition, regressive cellular processes with pathological changes indicating apoptosis or necrosis and massively pigmented cells were detected in the conditions where EP was applied with either calcium or bleomycin (Figure 3C–P and Figure 4). Furthermore, a dissociation of the tumor cells and lower adhesion was observed particularly in the core of the grafts, with signs of migration of pigmented tumor cells in the proximal area of the CAM (Figure 3D,F,H,L,N,P). The positive invasion was predominantly detected in the conditions with EP (Figure 3E,J,M,P). Moreover, the formation of tumor cell aggregates distributed predominantly in the periphery of the tumor was noted for the UPMM3 tumors (Figure 3F,H,K,L).

As observed on the CAM surface during the development period, UPMD2 cells built intensely pigmented dark brown nodules, whereas UPMM3 cells formed whitish light brown tumors (Figure 3A,C,E,G,H,L,N). The presence of intense pigmentation in the histological assessment was more prominent in the UPMD2 cell line in the ECT group with bleomycin compared to calcium and showed a dose-dependent effect (Figure 4). The apparent response in all tested agents in combination with EP and the absence of significant differences between the used concentrations of bleomycin as well as calcium underline the enhanced efficacy of the applied drugs even in lower dosage due to the electropermeabilization.

### 2.3. Immunofluorescence

For the evaluation of the viability of the grafts as well as the efficacy of the tested treatment conditions and modalities, various immunofluorescence markers were selected for detection. Slides stained with omission of the primary antibody served as negative controls of the examined marker. DAPI was used for counterstaining and revealed a reduced signal in the conditions combined with EP, depicting round and homogenously stained nuclei for the untreated group and a dose-dependent altered staining pattern with increased nuclear fragmentation in the ECT and CaEP group.

#### 2.3.1. Quantitative Analysis of Proliferation with Anti-Ki67 Antibody

**Figure 5:**
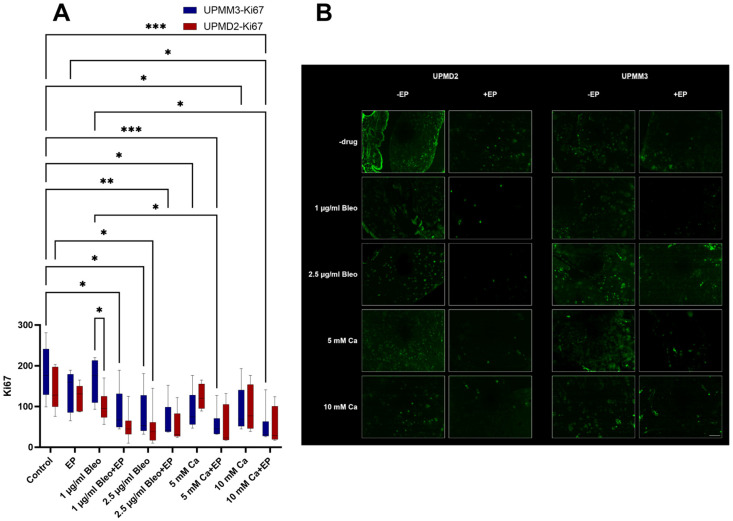
Quantitative evaluation of the Ki67 marker for the assessment of cell proliferation in the UPMM3 and UPMM2 cell xenografts, counts for positive cells per calculated area (**A**). A statistically significant decrease was observed in the groups treated with both EP and a drug agent. Higher doses of bleomycin or calcium chloride alone were required for a similar effect. Statistical analysis was performed using a two-way ANOVA and Tukey’s multiple comparisons test. Significance levels are indicated with * *p* < 0.05, ** *p* < 0.01, *** *p* < 0.001. Tumor images of immunofluorescence with Ki67 conjugated with Alexa Fluor 488 pro experimental treatment condition and cell line, 20× magnification (**B**). A prominent reduction of signal and therefore cell count for proliferation is noted between the applied agents with and without concomitant EP. A dose-dependent effect is documented for both tested drug agents. Scale bar 100 µm.

Immunofluorescence images were obtained after photodocumentation with a camera attached to a fluorescent microscope. The quantitative evaluation of Ki67-positive cells was carried out using Image J (Figure 5A,B). Ki67 is a protein that is present in the nucleus of cells during active phases of the cell cycle: G1 (first gap phase), S (synthesis phase), G2 (second gap phase), and mitosis. It is absent in resting cells (G0 phase). It therefore can provide information about the proliferative activity of cells [37]. The detection of expression of Ki67 comprises a well-established and reliable method to quantify cellular proliferation.

EP alone did not show to significantly influence the cell proliferation in the UPMM3 and UPMD2 cell xenografts (*p* = 0.8). Similarly, 1 µg/mL bleomycin alone did not lead to a statistically significant proliferative reduction, but a prominent difference between the two cell lines could be noted, with lower values for the UPMD2 cells. A variation of counts for the two tested cell lines without statistical significance was further observed in the group treated solely with 5 mM Ca with a higher proliferative index for the UPMD2 tumors (Figure 5A). These results suggest a higher sensitivity for bleomycin and a potential resistance for calcium in the UPMD2 cell xenografts. Doses of 2.5 µg/mL bleomycin and 10 mM Ca alone induced a prominent decrease in the proliferation rate intratumorally in both cell lines. No significant differences were detected in the conditions with EP and bleomycin or calcium between the different tested concentrations, postulating the prospective achievement of an equal reduction in the cell proliferation, while lower concentrations of the applied drug are required.

#### 2.3.2. Quantitative Analysis of Vascularization with Anti-CD31 Antibody

**Figure 6:**
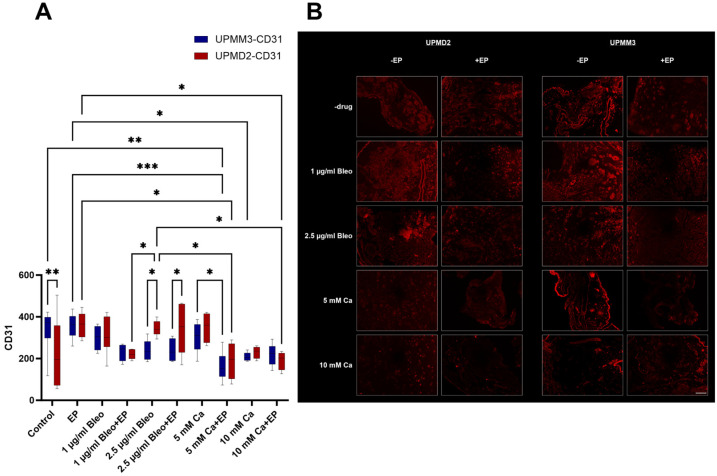
Quantitative evaluation of the CD31 marker for the assessment of the angiogenesis and vascularization in the UPMM3 and UPMM2 cell xenografts; counts for positive cells per calculated area (**A**). A statistically significant decrease was observed in the group treated with 5 mM Ca and EP predominantly for the UPMM3 tumors. Higher sensitivity levels to calcium in comparison to bleomycin were documented regarding the detection of CD31-positive cells. Statistical analysis was performed using a two-way ANOVA and Tukey’s multiple comparisons test. Significance levels are indicated with * *p* < 0.05, ** *p* < 0.01, *** *p* < 0.001. Tumor images of immunofluorescence with CD31 conjugated with Alexa Fluor 594 pro experimental treatment condition and cell line, 20× magnification (**B**). A prominent reduction of signal and therefore cell count for angiogenesis was noted between the applied agents with and without concomitant EP, especially in the settings where calcium chloride was applied. Scale bar 100 µm.

CD31 (platelet endothelial cell adhesion molecule-1, PECAM-1) was selected in the current experimental set-up for the assessment of vascularization in the treated and untreated tumors to evaluate the efficacy of the applied therapeutic modalities. It is expressed ubiquitously within the vascular compartment and is located mainly at junctions between adjacent cells. Furthermore, it is a molecule with various functions, highlighting its importance in multiple physiological and pathological processes, including immune responses, apoptosis, angiogenesis, vascular biology, inflammation, integrin-mediated cell adhesion, and thrombosis. It is one of the key regulatory molecules in the vascular system and angiogenesis, since PECAM-1 enables the formation of new blood vessels through the cell–cell adhesion [38]. The generated immunofluorescence images were assessed for cell counting and statistical analysis (Figure 6A,B).

A significant reduction in the CD31 signal and therefore in the vascularization in comparison to the control group was exclusively documented for the tumors treated with 5 mM Ca combined with EP for the UPMM3 cell line (*p* = 0.0023). For the UPMD2 tumors, an apparent reduction was similarly documented with a statistically significant difference in comparison to the EP group (*p* = 0.034) but not to the control group for the same treatment condition. Only UPMM3 nodules treated with 10 mM Ca alone showed a significant difference in the detection of CD31 in comparison to EP alone (*p* = 0.043), whereas a prominent but not significant reduction was noted in UPMD2 nodules for this condition. A statistically significant decrease in the CD31-positive cell count was solely documented for UPMD2 tumors when treated with the combined application of 10 mM Ca and EP in comparison to EP alone (*p* = 0.036). A higher sensitivity of the UPMM3 cell line was discovered regarding the effect on angiogenesis and vascularization after treatment with 2.5 µg/mL bleomycin alone (*p* = 0.013) or with EP (*p* = 0.027). The described discrepancy could not be detected between the tested cell lines for the combination of 1 µg/mL bleomycin alone or with EP. The most prominent decline in CD31 fluorescent-positive cells could be achieved with ECT with 1 µg/mL bleomycin among all tested bleomycin conditions. Moreover, EP, 1 or 2.5 µg/mL bleomycin, and 5 mM Ca alone could not reproduce a similar effect on the detection of CD31 in the immunofluorescent staining (Figure 6A).

#### 2.3.3. Quantitative Analysis of Apoptosis with Anti-Caspase-3 Antibody

**Figure 7:**
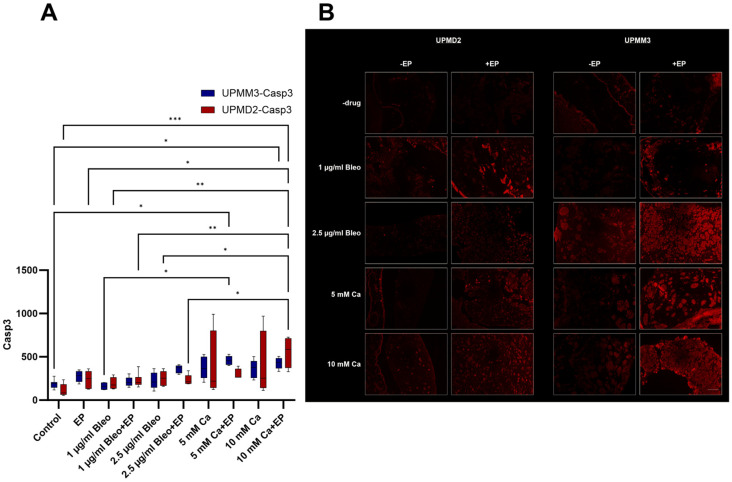
Quantitative evaluation of the caspase-3 marker for the assessment of the apoptotic rate in the UPMM3 and UPMM2 cell xenografts among the various treatment settings; counts for positive cells per calculated area (**A**). A statistically significant increase was observed in the group treated with 5 mM Ca and EP for the UPMD2 tumors and after application of 5 mM Ca and EP for the UPMM3 xenografts. Higher sensitivity levels to calcium in comparison to bleomycin were documented regarding the induction of apoptosis. Statistical analysis was performed using a two-way ANOVA and Tukey’s multiple comparisons test. Significance levels are indicated with * *p* < 0.05, ** *p* < 0.01, *** *p* < 0.001. Tumor images of immunofluorescence with caspase-3 conjugated with Alexa Fluor 594 pro experimental treatment condition and cell line, 20× magnification (**B**). A prominent increase of signal and therefore of the apoptotic rate was noted between the applied agents with and without concomitant EP, especially in the settings where calcium chloride was applied. Scale bar 100 µm.

The caspase-3 protein is a member of the cysteine-aspartic acid protease (caspase) family [39]. Caspase-3 is activated in the apoptotic cell both by the death receptor (extrinsic) and mitochondrial (intrinsic) routes [40]. The pro-enzymatic feature of caspase-3 is essential, and in case of regulation disturbance, caspase activity would trigger cell death indiscriminately [41]. The caspase-3 inactive precursors have virtually no executional function until they are cleaved by an initiator caspase after apoptotic signaling events have occurred. Therefore, caspase-3 constitutes a suitable option for the evaluation of apoptotic induction after antitumoral therapeutical modalities via immunofluorescence and subsequent quantification (Figure 7A,B).

A significant increase of the apoptotic rate could be achieved in the UPMD2 xenografts via the application of 10 mM Ca and EP (*p* = 0.0001). A similar effect was evident for the UPMM3 tumors after treatment with 5 mM Ca combined with EP in comparison to the control group (*p* = 0.014). A prominent increase without statistical significance in comparison to the other tested groups was additionally observed in the generated nodules from both cell lines after injection of both 5 and 10 mM Ca. Treatment settings with bleomycin induced solely a slight dose-dependent increase of apoptosis, especially for the UPMM3 tumors after ECT with 2.5 µg/mL bleomycin (Figure 7A).

#### 2.3.4. Quantitative Analysis of Melanoma Cells with Anti-Melanoma Antibody-Melanoma-Mix

**Figure 8:**
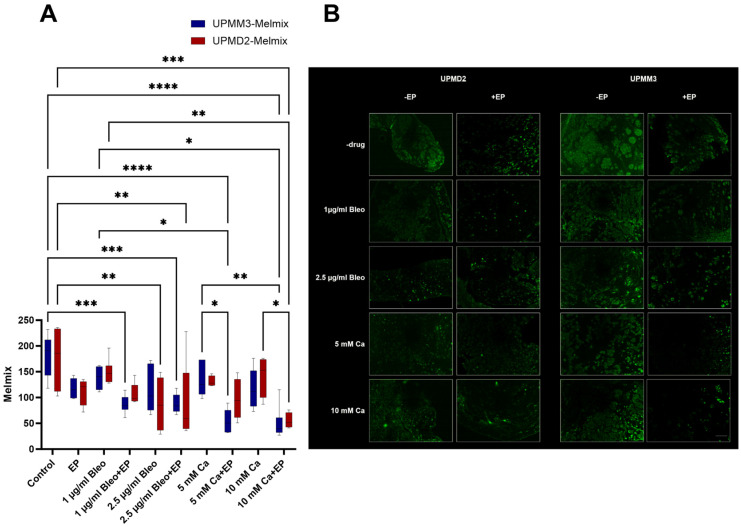
Quantitative evaluation of the melanoma markers for the assessment of the melanoma cell count in the UPMM3 and UPMM2 cell xenografts among the various treatment settings; counts for positive cells per calculated area (**A**). A statistically significant decrease was observed in the group treated with EP and 10 mM Ca as well as 2.5 µg/mL bleomycin for both cell lines and after application of EP and 5 mM Ca as well as 1 µg/mL bleomycin for the UPMM3 xenografts. Higher sensitivity levels to calcium in comparison to bleomycin were documented regarding the reduction of melanoma cells predominantly in the UPMM3 tumors. Statistical analysis was performed using a two-way ANOVA and Tukey’s multiple comparisons test. Significance levels are indicated with * *p* < 0.05, ** *p* < 0.01, *** *p* < 0.001, **** *p* < 0.0001. Tumor images of immunofluorescence with HMB45, M2-7C10, M2-9E3 conjugated with Alexa Fluor 488 pro experimental treatment condition, and cell line, 20× magnification (**B**). A prominent decrease in signal and therefore in the melanoma cell count was documented between the applied agents with and without concomitant EP, with a more prominent effect for the UPMM3 xenografts. Scale bar 100 µm.

A cocktail of recombinant multiclonal anti-melanoma antibody consisting of HMB45 and two different clones of MART-1 (M2-7C10, M2-9E3) was selected for the quantification of the viable melanoma cells in the examined tumors as well as their migration in the near proximity (Figure 8A,B). The MART-1/Melan-A antigen is thus a suitable marker for melanocytic tumors, predominantly melanomas, with the caveat that it is normally found in benign nevi as well. Despite its relatively high sensitivity, HMB-45 can only be detected in a portion of melanomas and is nonreactive with almost all non-melanoma human malignancies, with the exception of rare tumors showing evidence of melanogenesis [42].

A significant reduction of melanoma cell count was observed for both UPMM3 and UPMD2 xenografts after treatment with EP combined with either 10 mM Ca (*p* < 0.0001, UPMM3, *p* = 0.0002, UPMD2) or 2.5 µg/mL bleomycin (*p* = 0.001, UPMM3, *p* = 0.0093, UPMD2). A higher sensitivity was documented for the UPMM3 cell line since a significant decrease of melanoma-positive cells was detected for the combination of EP and administration of 1 µg/mL bleomycin (*p* = 0.0004) as well as 5 mM Ca (*p* < 0.0001). A statistically significant decrease in comparison to the control group was furthermore evident solely in the UPMD2 grafts after injection of 2.5 µg/mL bleomycin alone (*p* = 0.0023). EP, 1 µg/mL bleomycin, and 5 or 10 mM Ca alone did not induce a sufficient divergence to achieve a significant effect on the viability of melanoma cells (Figure 8A).

### 2.4. Image Analysis via Image J

The development of the tumors during the growth period was evaluated for each group on ED 7 and 18 based on the calculation of the cross-sectional area, the perimeter, as well as the mean gray value of the photographic images via image analysis with Image J before and after the applied therapy.

**Figure 9:**
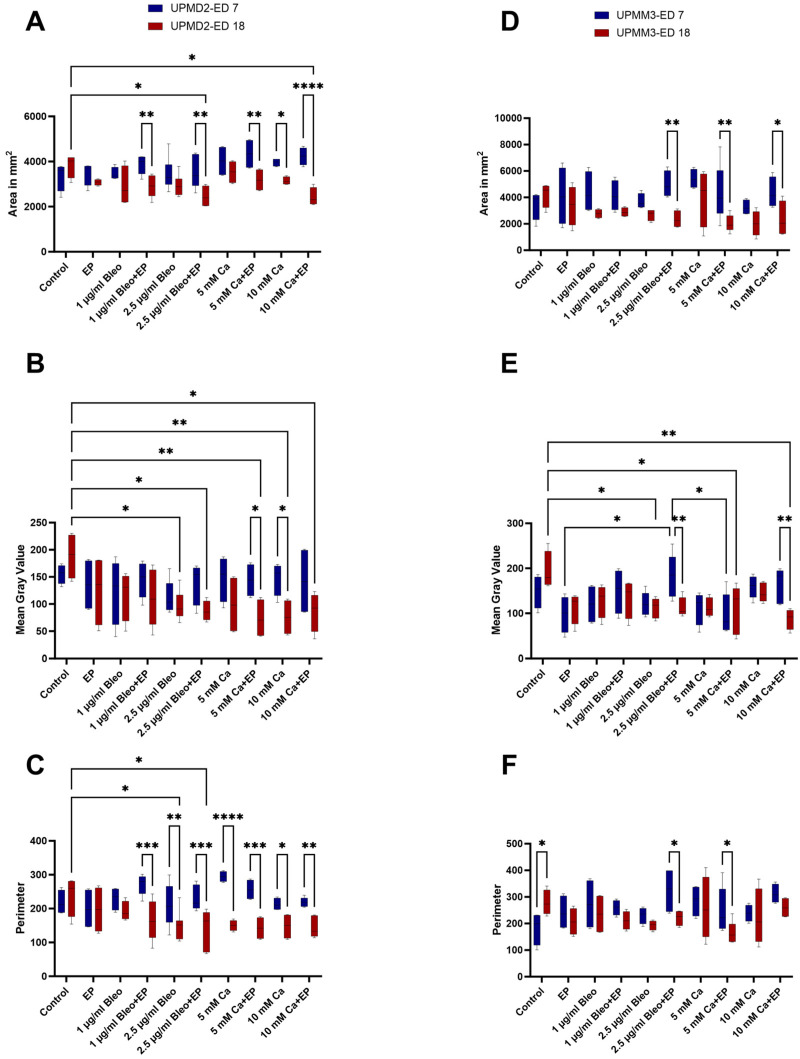
The cross-sectional area, mean gray value, as well as the perimeter of the implanted UPMD2 and UPMM3 UM xenografts on ED 7 and ED 18 via Image J 1.53t. (**A**,**D**): Comparison of the calculated cross-sectional area of the UPMD2 and UPMM3 grafts between the tested treatment conditions and during the growth period between ED 7 and 18. (**B**,**E**): Measurement of the mean gray value as an indicator of the tumor density of UPMD2 and UPMM3 tumors among the various applied therapeutic settings and between ED 7 and 18. (**C**,**F**): Calculation of the perimeter as a further size and distribution parameter and comparison of the effect after treatment with drug agents alone or in combination with EP between ED 7 and 18 for UPMD2 and UPMM3 xenografts. Statistical analysis was performed using a two-way ANOVA and Tukey’s multiple comparisons test. Significance levels are indicated with * *p* < 0.05, ** *p* < 0.01, *** *p* < 0.001, **** *p* < 0.0001.

The cross-sectional area was measured as an index of growth or regression after therapy to compare the effect of the tested modalities further quantitatively on the tumor size (Figure 9A,D). EP combined with 2.5 µg/mL bleomycin (*p* = 0.0023, UPMD2, *p* = 0.0036 UPMM3) as well as 5 (*p* = 0.0028, UPMD2, *p* = 0.0049, UPMM3) and 10 mM Ca (*p* < 0.0001, UPMD2, *p* = 0.024, UPMM3) induced a significant reduction of the calculated area between ED 7 and 18 for both UPMD2 and UPMM3 xenografts. UPMD2 tumors showed a similar sensitivity and tumor shrinkage after ECT with 1 µg/mL bleomycin (*p* = 0.0064). The tested drugs alone without EP induced only a slight size decrease without statistical significance.

Furthermore, the mean gray value was determined for all tested experimental settings, since it is indicative of the tumor density and represents the sum of the gray values of all the pixels in the selection divided by the number of pixels. UPMD2 tumors showed a significant reduction in the density after injection of 5 mM Ca and application of EP with 8 pulses and 1000 V/cm (*p* = 0.0165) and after 10 mM Ca alone (*p* = 0.0148). A prominent reduction in the MGV could be observed in the UPMM3 tumors after intratumoral administration of 10 mMCa with EP, but the drug alone did not lead to statistically significant changes (*p* = 0.0016). On the contrary, UPMM3 tumors showed a positive response after ECT with both doses of bleomycin, whereas only the combination with 2.5 µg/mL bleomycin resulted in a significant decrease in density (*p* = 0.0048) (Figure 9B,E).

For the identification of the shape and size distribution, the perimeter was additionally calculated and compared between ED 7 and 18 for both tested tumor types and treatment conditions (Figure 9C,F). A higher sensitivity was noted regarding the reduction of the perimeter in the UPMD2 tumors, which showed a great response after treatment with 2.5 µg/mL bleomycin and both doses of calcium alone as well as with all tested drug doses when combined with EP. On the contrary, on ED 18, UPMM3 displayed an exponential growth with significant difference to the perimeter and distribution of the tumor, in comparison to the pre-treatment period, when untreated (*p* = 0.0378). A significant reduction in the perimeter from 324.5 (±85.96 SD) mm to 221.25 (±28.59 SD) mm and from 248.6 (±87.46 SD) mm to 163 (±43.67 SD) mm was observed after application of EP with 2.5 µg/mL bleomycin or with 5 mM Ca accordingly.

## 3. Discussion

Although advances in the diagnosis and treatment of UM have improved the management of the disease, its estimated metastatic rate remains approximately 50% and is subsequently related to a highly dismal prognosis [43]. Nevertheless, there is still a restricted number of therapeutic options in the treatment of metastatic or unresectable UM, which constitutes a common consensus for a standardized therapy and the urgent development of novel adjuvant strategies. Preclinical evaluation of novel therapeutics and treatments for this disease have relied on in vitro assays and in vivo rodent studies, which demand time and costly resources, special training, and interfere with animal-welfare ethical issues. Translation into the clinic strongly relies on such models and has therefore been one of the hurdles in developing new effective modalities. We established a UM xenograft model based on the CAM assay using UPMD2 and UPMM3 cell lines to generate in vivo UM tumors and to examine the efficacy of CaEP in comparison to the ECT with bleomycin as a novel adjuvant treatment for UM.

References to the CAM assay as an alternative in vivo xenograft model for studying major hallmarks of cancer such as proliferation, angiogenesis, metastatic profile, and assessment of pharmacotherapy have exploded in the literature in the last decade since it complies with the 3R (replacement, reduction, refinement) principles [44]. It provides a unique biological microenvironment appropriate for cancer cells. Furthermore, the generated tumors after inoculation of the cancer cells resemble the tumor microenvironment composition, also consisting of cancer cells, extracellular matrix, collagen, stromal cells, and tumor vascularity [45,46]. The experiments are terminated before the development of neurological structures of the embryos associated with pain perception, and the CAM itself does not possess a nervous system. Therefore, studies involving the CAM are not considered to cause distress to the embryos and are exempt from approval by animal or ethics committees in the majority of countries in Europe [47]. As the CAM and the grafts are easily visualized and accessible, the administration of compounds is uncomplicated. Thus, the CAM assay can open new perspectives towards the preclinical evaluation of rare tumor entities and the improvement of selection of drug candidates, reducing the failure rate of anti-cancer drugs in clinical studies [48]. Moreover, it is a xenograft model, which is more time- and cost-effective and represents an efficient intermediate step between in vitro and in vivo studies. These tumors mimic, albeit partially, the native growth and histological features and can be used to assess responses to new treatments as an initial screening, focusing on delineating specific subtargets for further preclinical validation in rodent models. Likewise, the selection of the tested drugs and their concentrations in the current experiment was based on the results of earlier projects with ECT and CaEP. Due to its natural immunodeficiency until day 11, the CAM accepts transplants from various tissue types and species without immune reaction. T cells can be first detected at day 11, and B cells at day 12, and the chicken embryos reach their full immunocompetency by day 18 [49,50]. Its rich vascularization allows the rapid growth of the grafts, and the CAM vessels tend to attach and grow into the tumors placed on its surface. The formation of a vascular star around and often of a vascular ring at the basis of the tumor was considered a sign of successful implantation and was usually seen before the end of the first week after grafting. Compared with other mammalian models, where tumor growth takes between 3 and 6 weeks, the tumor development in the CAM assay requires between 2 and 5 days after tumor cell transplantation [44].

The short timeframe of the assay can potentially be compensated through reimplantation of the grafts and therefore prolongation of the observation period. Cancer xenograft models derived from cell lines are known to suffer drawbacks of in vitro culturing prior to implantation, including the loss of biological complexity under culture conditions. Cells might possess inherent demerits after passages with acquisition of molecular alternations and genetic instabilities during cell culture adaptation, which may lead to varied experimental results between passages [51]. The presented xenograft model represents a valid model for the analysis of many aspects of UM biology. However, continued baseline analyses are necessary using a larger panel of UM cell lines or even patient-derived grafts covering the spectrum of genomic and phenotypic alterations observed in patients [52,53].

ECT is a promising technique, which has been proven to be an effective alternative in the palliative management of unresectable recurrent solid tumors or the locoregional control of disseminated lesions with a response rate of approximately 80–90%, also resulting in amelioration of quality of life for the patients [27]. The cell membrane represents a dielectric separating the cytoplasm and the external medium, as conductive media. When the membrane is subjected to an external electric field, the cell behaves as a closed capacitor. As a result, the electric field induces a size- and position-dependent transmembrane potential, which superimposes to the resting potential. If the induced transmembrane voltage reaches a certain critical level, this potential can lead to membrane permeabilization or poration [54]. With ECT, electroporation is reversible, and the disturbances of cell membrane properties are temporary. ECT was initially conducted with bleomycin for proof of concept, where the cytotoxicity of the drug was enhanced 700-fold after EP, compared to the drug alone [55]. ECT consists of an alternate approach to traditional chemotherapy by enhancing the potency of various other cytotoxic drugs such as cisplatin and constitutes an established treatment of advanced tumor disease such as cutaneous melanoma [56,57].

Various pathomechanisms have been postulated for the antitumoral efficacy of ECT. The accumulation of otherwise non-permeant chemotherapeutic agents and their locally multifold enhanced cytotoxic effect induce cell death mitosis. EP can cause cell death, inducing apoptosis, but other mechanisms, such as necrosis or immunogenic death, can be present, depending on cell and tissue type and also on treatment zones. The reduction of cell proliferation in our study was significant for both cell lines in all conditions combined with EP regardless of the applied drug concentration. An increase in the apoptosis was observed for the UPMD2 and UPMM3 tumors following ECT with 2.5 µg/mL bleomycin as well as after CaEP with both 5 and 10 mM. Another effect was the release of intact tumor antigens due to substance shedding by the damaged cells. Released tumor antigens are exposed to the immune system of the patients and trigger a tumor antigen-directed immune response [58,59]. The recruitment of various components of the immune system may elicit a systemic reaction even against distant metastases, constituting the abscopal effect [60,61]. The vascular lock due to the induced vasoconstriction of arterioles and the interstitial edema of endothelial structures leads to the reduction of blood supply to the tumor and allows the prolongation of the drug retaining time [62,63]. The effect was also instantly visible in our experimental setting after application of the EP on the CAM with a temporal receding of the proximal vessels, occasionally accompanied by fine hemorrhage. The vascularization of the grafts was histologically assessed and resulted in significant differences between the two xenograft types with significantly higher values, especially in the conditions with 2.5 µg/mL bleomycin and lower values in the control group for the UPMD2 tumors. The absence of drastic changes after treatment regarding vascularization in the UPMM3 xenografts may be attributed to the genetic characteristics of the cell line, since it is derived from a metastatic tumor with monosomy 3, and also to the reduced expression of functionally related components of the ribosomal translation machinery [35]. This imbalance could explain the poor proliferative behavior and vascular response of the UPMM3 generated grafts as well as the difficulties in their maintenance. The values for proliferation showed a lower tendency for all tested conditions in the UPMM3 cell line. Furthermore, a higher amount of eggs and implantations was necessary to achieve the same number of successful grafts and growth in comparison to the UPMD2 tumors. In addition, the more intense pigmentation of the UPMD2 xenografts facilitated their evaluation and the experimental procedure.

In regards to the field of ocular oncology, its use has been described for the clinical treatment of large ocular basal carcinomas in three patients with periocular lesions and significant comorbidities that did not allow a wide surgical resection [64]. Furthermore, ECT was demonstrated to be a feasible locoregional treatment and beneficial non-invasive option with pleasing aesthetic results in a case with a multirecurrent dermatofibrosarcoma protuberans of the orbital margin [65]. The use of ECT with intravenous administration of bleomycin was reported in a single case of ocular melanoma in a dog with complete tumor remission [66]. A needle electrode was used for the administration of an electric field of 1.300 V/cm, eight electric pulses, and a frequency of 5 Hz, which was positioned parallel to the retina to prevent dislocation. The homogeneous distribution of the electric field between the electrode and the treated area was ensured through the application of an ultrasound gel. Corneal covering and a tarsorrhaphy for 15 days were conducted following ECT. In the follow-up after 7 months, no signs of recurrence could be detected. In another experimental setting, the effect of ECT was investigated in vitro in four primary and metastatic UM cell lines, Mel 270, UM 92.1, OMM-1, OMM-2.5, and showed a higher resistance to cisplatin in comparison to bleomycin, although all cell lines demonstrated a greater response to and high efficacy of ECT than the highest concentration of the antineoplastic agent alone [67]. Moreover, the application of 2.5 µg/mL bleomycin in combination with EP 750 V/cm was explored by the same group in 3D spheroids from primary and metastatic cell lines, MP 46, UM 92.1, Mel 260 MM 28, and OMM-1, as well as during single attempts of implantation in the CAM assay. They showed a positive response in comparison to monotherapy but varying results depending on the origin and type of the cells [68]. This observation is confirmed by our results, where a variable sensitivity was documented for the different applied settings among the two tested cell lines with more prominent or significant differences between the UPMD2 and UPMM3 xenografts in the assessment of proliferation and vascularization. Our group reported the design of a customized electrode to facilitate the conduction of ECT in uveal and conjunctival melanoma spheroids. The results supported a significant reduction of viability and growth 3–7 days after ECT with 750 V/cm, 8 pulses of 100 µs duration, 5 Hz, and 2.5 µg/mL bleomycin for the tested cell lines. After prior optimization, we selected the electric pulse setting of 8 pulses, 1000 V/cm, and 5 Hz as suitable for the applied drugs and their concentrations for the present study. Furthermore, the customized electrode with 1 mm thickness, 4 mm width, 8 mm length, and adjustable gap between the two electrodes of approximately 3–5 mm was optimized and used in the current experiment. The radiosensitizing effect of concomitant ECT of primary and radioresistant UM cell lines UPMD2, UPMM3, UM92.1, and Mel270 with irradiation has also been postulated in the literature [69]. ECT is also suggested as an alternative for radioresistant head and neck squamous cell carcinomas [70]. The administration of bleomycin intravenously or intratumorally have been compared and seem to be equally effective at delivering similar response rates [27].

The mechanism behind CaEP is highly complex, since calcium is an essential messenger involved in the regulation of various cellular processes. Calcium is mainly stored in the endoplasmic and in the sarcoplasmic reticulum and in mitochondria. An increased mitochondrial calcium concentration, as it occurs during CaEP, can modulate mitochondrial metabolism by increasing the ATP production, but it can also trigger cell death, apoptosis, or necrosis [71,72]. The activation cascade may further induce the activation of proteases and lipases or generation of reactive oxygen species [32]. The increased accumulation in the mitochondria may likely limit or destroy the mitochondrial respiration and thereby the ATP production or induce ATP depletion due to increased consumption to excrete calcium excess. Apart from the increased calcium uptake, the cell death has been shown to be by apoptosis or necrosis, depending on the cell type, calcium concentration, time of measurement, as well as examination method [73,74]. Through variations in the permeabilization and the membrane repair capacity, the recovery of the intracellular calcium level after treatment with CaEP may differ between cell types [75]. In the study of Falk et al., with the activation of immune stimulators and the increased release of the high mobility group box 1 protein (HMGB1), a damage-associated molecular pattern molecule important in immunogenic cell death indicates immunogenic cell death [76]. Therefore, CaEP could be used as an in situ vaccination for combination with immunomodulatory therapies. Proteins involved in regulating calcium signals are often remodeled in cancer cells compared to normal cells to sustain proliferation and avoid cell death. Calcium channels, pumps, and exchangers show differences in their expression, localization, and activity among cancerous and healthy cells, including the localization of phosphatidylserine in the outer cell membrane leaflet of malignant cells [77,78]. Moreover, CaEP, similar to ECT, shows a difference in sensitivity between normal and malignant cells in vitro and in vivo, which might partly be attributed to the forementioned changes in expression but also to differences in membrane repair capacities [75,79,80]. The apoptotic effect in our study was greater for both cell lines following ECT with 2.5 µg/mL bleomycin and CaEP with 5 or 10 mM Ca in comparison to monotherapy, whereas a statistically significant increase in the apoptosis was only observed after CaEP with 5 mM Ca in the UPMM3 and 10 mM Ca in the UPMD2 xenografts. Furthermore, the UPMD2 tumors revealed higher apoptotic values, underlying the varying sensitivity to the CaEP between various cell types.

Furthermore, Kraemer et al. investigated calcium electroporation in 2D and 3D UM cell spheroids, deriving from UM92.1, Mel270, UPMD2, and UPMM3. The authors demonstrated a significant decrease in the ATP for all tested cell lines after CaEP and ECT with bleomycin, suggesting a dose-dependent ATP depletion with a wide range of sensitivity among the tested cell lines [81]. The specific growth rate was significantly reduced in the UM92.1 after CaEP with 0.5 and 1.1 mg/mL Ca, whereas the same effect in the Mel270 could only be achieved with administration of 1.1 mg/mL Ca. Our analysis of the size parameters after the tumor harvesting revealed the size reduction in all tested conditions involving combined therapy with EP in both tumor types, with a more apparent effect following ECT with 2.5 µg/mL bleomycin and CaEP with 5 or 10 mM Ca. The histological assessment confirmed these results with a prominent size reduction after CaEP with 5 mM in UPMD2 as well as with 5 and 10 mMCa in UPMM3 xenografts. The aforementioned study reported that Mel270 showed signs of lower adhesion and density three days after therapy, while the UPMD2 and UPMM3 demonstrated signs of detachment at a later point, after seven days. In the current investigation, the tested cell lines were selected based on prior findings of our group in vitro and on the intriguing potential variations in response due to their divergent genetic characteristics. UPMM3 cells display poor proliferation and a higher tendency for invasion. They derive from a tumor with monosomy 3 and are therefore considered representatives of higher metastatic potency and of aggressive melanoma cells. UPMD2 cells are generated from a primary uveal melanoma tumor with disomy 3 and show higher proliferation rates, which could explain the more robust and pigmented growth of the UPMD2 xenografts. We also noticed dissociation of the tumor core in the tumors treated with EP conditions. This could partially be attributed to the intratumoral drug injection and could potentially represent an artefact due to external manipulation, since this effect was furthermore less evident in the control group. Additional lower adhesion of the cells with positive migration in the proximal area of the CAM was also noted for the treatment conditions combined with EP. Furthermore, the presence of intense pigmentation was more common in the histological evaluation of the UPMD2 tumors, which displayed a dose-dependent induction predominantly by bleomycin. An overall positive response regarding the size parameters and the tumor configuration was observed in both cell lines for the combined treatment in comparison to monotherapy. The immunofluorescence of the samples also demonstrated lower levels of the melanoma-mix staining in the UPMM3 with overall reduction of values and a significant effect following ECT with 2.5 µg/mL bleomycin and after CaEP with 5 and 10 mM Ca for both cell lines. The density of the tumors based on the image analysis showed a reduction in density for both UPMM3 and UPMD2 xenografts with more prominent differences during the experimental phase for the UPMD2 cell line and specifically for EP combined with 2.5 µg/mL bleomycin as well as 5 and 10 mM Ca. Maniotis et al. reported that UPMM3 cells were able to form vasculogenic cords, which is a postulated positive indicator for tumor cell aggressiveness, which was absent in the UPMD2 cells [82]. This finding could explain the more prominent changes in the tumor density or the more apparent dissociation and the differences in the tumor response of the UPMD2 xenografts following treatment.

The preselection of subsets of patients and efficient treatment regimens allows the development of precision medicine in oncology with the constant goal of reducing morbidity and mortality. The application of EP-based modalities for the treatment of UM may represent a feasible alternative, inducing a positive tumor response and allowing the administration of lower concentrations of cytotoxic drugs or non-toxic agents, such as calcium with minor adverse effects. The mouse model remains irreplaceable in translational medicine prior to clinical application to patients. The presented in vivo UM model may contribute to the preselection of the optimal experimental settings prior to application to rodent models, potentially accelerating the establishment of new effective therapeutic strategies. Thus, electroporation constitutes a promising novel tool for optimizing local tumor control in UM. The conception and design of new electrodes, specifically for the eye bulb, would further facilitate the clinical application. It could potentially represent a neoadjuvant treatment option for downstaging more extensive local invasive UM, achieving a higher possibility of local tumor control and eye salvage. Its application in metastatic lesions could potentially contribute to the amelioration of quality of life and to prolongation of the life expectancy of UM patients.

## 4. Materials and Methods

### 4.1. Chicken Chorioallantoic Membrane Assay

To avoid contamination, 50% ethanol was used to clean fertilized white Lohmann chicken eggs. At a temperature of 37.5 °C and a humidity of approximately 60–70%, the eggs were positioned with an upright orientation in an incubator to induce embryogenesis (Bruja 3000 digital, Siepmann, Germany and Mini Pro 147, Maino, Italy). After optimization of our protocols, as discussed in our previously published studies, the CAM was lowered by removing 4–8 mL albumin on ED 5 with a 10 mL sterile syringe and a 20-gauge safety butterfly cannula (Safety-Multifly Needle, Sarstedt, Nümbrecht, Germany). A surgical tape was used to reseal the puncture site (3M Micropore surgical tape, Saint Paul, MN, USA). Under aseptic conditions, a window was carefully opened in the eggshell on ED 6 without damaging the underlying embryonic structures to facilitate the implantation of the generated tumor cell pellets. Subsequently, Parafilm was placed over the opening (Bemis Company Inc., Neenah, WI, USA) and the eggs were positioned back in the incubator until the implantation on ED 7.

### 4.2. Cell Lines and Culture Conditions

The experiments on the CAM were conducted using two cell lines, UPMD2 (accession number: CVCL_C298) and UPMM3 (accession number: CVCL_C295) and were provided by M. Zeschnigk (Institute of Human Genetics, University Hospital Essen, Essen, Germany). All cell lines were tested for authentication via Short Tandem Repeat (STR) before initiation of the experiments and were mycoplasma free at the time of experimentation. The cells were grown to confluency in cell culture flasks in a Ham/F12 medium (PAN-Biotech GmbH, Aidenbach, Germany) supplemented with 10% fetal calf serum and 1% penicillin-streptomycin (5000 U/mL) (PAN-Biotech GmbH, Aidenbach, Germany), which was exchanged two times per week. The cells were cultured in a humidified atmosphere containing 5% CO_2_ at 37 °C.

### 4.3. Uveal Melanoma Xenografts

**Figure 10:**
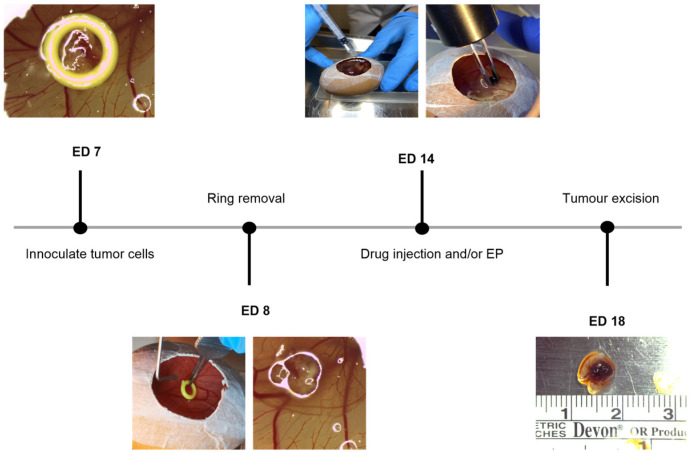
Corresponding timeline of the experimental procedure starting on ED 7 with the inoculation of 1 × 10^6^ cells of each cell line with Matrigel into a ring and onto the CAM, followed by the ring removal on ED 8, subsequently treating the generated UPMM3 and UPMD2 xenografts on the CAM surface with either EP or injection of bleomycin or calcium chloride alone or in combination with EP. On ED 18 the tumors were excised and the embryos sacrificed.

On the implantation day, ED 7, the UPMD2 and UPMM3 cells were gently washed with PBS and after its aspiration, they were incubated in trypsin for approximately two minutes. The digestion was stopped by adding medium, and the cells were separated and washed off the flask surface via resuspension before transfer in a 15 mL conical tube. Subsequently, 10 µL of the cell suspension was mixed with 10 µL Tryptan blue, and 10 µL of the mixture was applied in the counting surface of the cell counter (CellDrop FL, Fluorescence counter, DeNovix, Wilmington, Delaware, USA) to define the necessary volume for the required cell number pro pellet. The cells were spun at 500 rpm at 4 °C or room temperature for 5 min, and the supernatant was decanted from each centrifuged tube. Each pellet of 1 × 10^6^ cells was resuspended for both cell lines with 30 µL Matrigel (Corning Life Sciences, Corning, NY, USA). Prior to implantation, one prominent vessel was gently lacerated in the proximity of a vascular bifurcation, selected as the graft site. A sharp debridement spoon or a needle was used to induce a small bleed to enhance engraftment of the tumor cells. Plastic rings, created by cutting the tip of inoculating loops (Ino-Loop; Simport Scientific Inc., Saint-Mathieu-de-Beloeil, QC, Canada), were slightly taped to the periderm at the implantation site on ED 7. The generated tumor pellets coated with Matrigel were then carefully pipetted and placed in the middle of the ring. The ring was carefully removed on the following day. The tumor nodule formation was observed until ED 14, where the treatment of the grafts was conducted with either injection of the drug or combined with EP (Figure 10).

### 4.4. Treatment Conditions with Electroporation in Combination with Bleomycin or Calcium Chloride

Tumor grafts were treated either with 1 or 2.5 µg/mL bleomycin sulfate (bleomycin sulfate from *Streptomyces verticillus*, 1.5–2.0 units/mg solid, Sigma, St. Louis, MO, USA) alone or with 5 or 10 mM calcium chloride (calcium chloride dihydrate, Biochemica, Applichem GmbH, Darmstadt, Germany) alone, or in combination with EP via high-voltage electrical pulses using a voltage pulse generator (Cliniporator, IGEA S.p.A., Carpi, Italy). A further group consisted of tumor xenografts, which underwent solely EP. As a negative control, a further sample of tumor implants remained untreated. The EP was conducted with a customized two parallel aluminum electrode. The diameter of the electrodes is 1 mm, the gap between the two electrodes is 4 mm, and the length of each electrode is 8 mm. The administration of the tested agents, bleomycin and calcium, was carried out with an intratumoral injection of a 50 µL volume prior to EP in the according groups. Bleomycin remains the drug of choice and is well established for ECT, whereas the combination with calcium represents a novel treatment option due to the induced changes in calcium homeostasis in cancer cells [28,34,55,58,74,78,80]. The electrodes were placed directly on the CAM surface, and the tumor grafts were positioned between the two parallel aluminum rods. Subsequently, 8 pulses of 100 µs pulse duration, 5 Hz repetition frequency, and 1000 V/cm pulse strength were applied. The specific electroporation setting was selected after previous investigations of our group in vitro. The experiments were performed in four to nine independent biological replicates on different dates. The grown tumor nodules were surgically harvested on ED 18 (Figure 10). The embryos were sacrificed by decapitation. The lower CAM was inspected and microscopically examined for signs of tumor growth and pigmentation.

### 4.5. Assessment Assays

#### 4.5.1. Characterization of the Tumor Xenografts

The tumor grafts were monitored by photodocumentation on the CAM as well as after excision with a digital microscope camera (Leica M80, Leica IC80 HD, Leica Biosystems, Nußloch, Germany). The length and width of the tumor grafts were measured with a surgical marker ruler.

#### 4.5.2. Histology

After excision, the tumors were directly transferred into a plastic cassette and then kept in buffered formalin (Histofix 4%, Roth, Karlsruhe, Germany) for 24 h. Prior to dehydration with graded alcohols and xylene, the samples were incubated in PBS. All tumor specimens were embedded in paraffin at 58 °C prior to paraffin sectioning with 5 µm thickness with a rotary microtome (Reichert Jung 2040 Microtome Rotor Slicer, Cambridge Scientific Instruments GmbH, London, UK). Subsequently, the paraffin sectioned ribbons were transferred in a 36 °C water bath and were carefully placed onto histological slides after unfolding. The sections were left to dry at room temperature overnight. Furthermore, the deparaffination and rehydration of the slides was carried out before staining. Hematoxylin and eosin staining was performed for further morphological and cytological assessment. Furthermore, size characteristics, integration in the surrounding CAM tissue, vascular supply, and migration of tumor cells in the proximal structures were examined in the stained sections.

#### 4.5.3. Immunofluorescence

For the evaluation of vitality, proliferation, expansion, vascularization, and apoptosis, immunofluorescence staining of the specimens was performed. The sections were deparaffinized and rehydrated. The slides were incubated in PBS at room temperature for 10 min in cuvette. A universal HIER antigen retrieval reagent (Abcam, Cambridge, UK) was diluted 1:10 with distilled water, and the buffer solution was heated to 95 °C in cuvettes in a water bath. The slides were then placed into the cuvettes and incubated for 20 min. After reaching room temperature, they were rinsed with PBS twice, incubated with the blocking buffer (3%BSA in PBS) for 30 min, and drained. Furthermore, the primary antibodies were diluted and applied on the samples for a 2 h incubation. After drainage of the antibody solution, the slides were washed thrice in wash buffer. The secondary antibodies were applied for incubation for 30 min, and the slides were protected from light from this time point. The antibody solution was removed, then the slides were washed thrice and were mounted directly with anti-fade mounting medium (Invitrogen, Waltham, MA, USA). Stained specimens were visualized using an Olympus BX51 fluorescence microscope. In order to identify and mark human UM cells, a cocktail of mouse anti-HMB45, anti-M2-7C10, and anti-M2-9E3 primary antibodies (Abcam, Cambridge, UK) was applied at a 1:100 dilution. As an apoptotic and vascularization marker, a rabbit IgG polyclonal anti-caspase 3 (Cell Signaling Technologies, Danvers, MA, USA) and anti-CD31 (Invitrogen, Waltham, MA, USA), and for the evaluation of proliferation and viability a mouse anti-Ki67 primary antibody (Cell Signaling Technologies, Danvers, MA, USA), were used at a 1:400, 1:50, and 1:100 dilution accordingly. The selected secondary antibodies in combination with the nuclei marker DAPI (4′,6-diamidino-2-phenylindole) (Carls Roth, Karlsruhe, Germany) were goat anti-mouse Alexa Fluor 488 and goat anti-rabbit Alexa 594 (Invitrogen, Waltham, MA, USA) at a 1:400 dilution.

#### 4.5.4. Image Analysis

Image J 1.53t (Dresden, Germany) is an open-source software, which was used for the assessment of the photographic, histological, and immunofluorescent images to ensure an advanced image processing and an appraisable image analysis. The mean gray value, the perimeter, and the cross-sectional area were evaluated to estimate and quantify parameters such as the mass, density, and distribution and alterations in size, morphology, and thickness during the development phase as well as due to the applied treatment modality. Furthermore, the expression of the applied markers for proliferation, apoptosis, vascularization, and melanoma could be quantitatively determined.

### 4.6. Statistical Analysis

To detect the differences between the experimental groups and to determine how the response varies between the two tested cell lines, a two-way ANOVA test was carried out. Due to the high number of groups and complex combinations, Tukey’s multiple comparisons test was additionally performed (GraphPad Prism 10.0.1 software, GraphPad Software Inc., San Diego, CA, USA) [83]. A value of *p* < 0.05 was considered statistically significant, and significance levels were indicated as * *p* ≤ 0.05, ** *p* ≤ 0.01, *** *p* ≤ 0.005, **** *p* ≤ 0.001.

### 4.7. Ethics Approval

The ethics committee of the medical faculty of the University Duisburg-Essen approved the study with the number 21-9959-BO. The research was performed in accordance with the Declaration of Helsinki and with relevant local guidelines and regulations.

## 5. Conclusions

The present study aims to examine the use of a CAM-based UM model with tumor grafts from UPMD2 and UPMM3 cell lines for preclinical evaluation of rare tumor entities as well as the improvement of selection of drug candidates and new effective therapeutic strategies for the UM. Furthermore, the investigation focused on the assessment of efficacy following CaEP in comparison to ECT with bleomycin as an effective adjuvant treatment modality for UM regarding the melanocytic growth and expansion of the generated tumors as well as their proliferative rate, apoptotic rate, and vascularization. A dose-dependent effect was predominantly observed for bleomycin, whereas a varying sensitivity between the tested UM xenografts was documented among the various CaEP conditions. Despite its limitations, especially the short lifespan, the chick CAM assay represents a valuable timely and low-cost intermediate in vivo xenograft model that does not require the approval of an ethics committee and could be utilized to bridge the in vitro–in vivo gap more efficiently, reducing the failure rate of anti-cancer drugs in clinical studies. Our in vivo results suggest that CaEP and ECT may constitute an effective alternative option for an adequate local tumor control, which could open new horizons in the adjuvant treatment of advanced UM, potentially contributing to the bulbus preservation and improvement of quality of life for the UM patients.

## Data Availability

The data presented in this study are available in this article.
